# Deep learning-based lesion subtyping and prediction of clinical outcomes in COVID-19 pneumonia using chest CT

**DOI:** 10.1038/s41598-022-13298-8

**Published:** 2022-06-07

**Authors:** David Bermejo-Peláez, Raúl San José Estépar, María Fernández-Velilla, Carmelo Palacios Miras, Guillermo Gallardo Madueño, Mariana Benegas, Carolina Gotera Rivera, Sandra Cuerpo, Miguel Luengo-Oroz, Jacobo Sellarés, Marcelo Sánchez, Gorka Bastarrika, German Peces Barba, Luis M. Seijo, María J. Ledesma-Carbayo

**Affiliations:** 1 Biomedical Image Technologies, ETSI Telecomunicación, Universidad Politécnica de Madrid, Av Complutense 30, 28040 Madrid, Spain; 2 CIBER-BBN, Madrid, Spain; 3 Applied Chest Imaging Laboratory, Brigham and Women’s Hospital, Boston, MA USA; 4 Hospital Universitario La Paz, Madrid, Spain; 5 Hospital Universitario Fundación Jiménez Díaz, Madrid, Spain; 6 Clínica Universidad de Navarra, Pamplona, Spain; 7 Hospital Clinic de Barcelona-IDIBPAS, Barcelona, Spain; 8 CIBER-ES, Madrid, Spain; 9 Spotlab, Madrid, Spain; 10 Universidad de Vic (UVIC), Vic, Spain

**Keywords:** Prognostic markers, Predictive markers, Biomedical engineering, Computer science, Respiratory tract diseases

## Abstract

The main objective of this work is to develop and evaluate an artificial intelligence system based on deep learning capable of automatically identifying, quantifying, and characterizing COVID-19 pneumonia patterns in order to assess disease severity and predict clinical outcomes, and to compare the prediction performance with respect to human reader severity assessment and whole lung radiomics. We propose a deep learning based scheme to automatically segment the different lesion subtypes in nonenhanced CT scans. The automatic lesion quantification was used to predict clinical outcomes. The proposed technique has been independently tested in a multicentric cohort of 103 patients, retrospectively collected between March and July of 2020. Segmentation of lesion subtypes was evaluated using both overlapping (Dice) and distance-based (Hausdorff and average surface) metrics, while the proposed system to predict clinically relevant outcomes was assessed using the area under the curve (AUC). Additionally, other metrics including sensitivity, specificity, positive predictive value and negative predictive value were estimated. 95% confidence intervals were properly calculated. The agreement between the automatic estimate of parenchymal damage (%) and the radiologists’ severity scoring was strong, with a Spearman correlation coefficient (R) of 0.83. The automatic quantification of lesion subtypes was able to predict patient mortality, admission to the Intensive Care Units (ICU) and need for mechanical ventilation with an AUC of 0.87, 0.73 and 0.68 respectively. The proposed artificial intelligence system enabled a better prediction of those clinically relevant outcomes when compared to the radiologists’ interpretation and to whole lung radiomics. In conclusion, deep learning lesion subtyping in COVID-19 pneumonia from noncontrast chest CT enables quantitative assessment of disease severity and better prediction of clinical outcomes with respect to whole lung radiomics or radiologists’ severity score.

## Introduction

Severe or even fatal respiratory diseases affect 17% to 29% of hospitalized patients with COVID-19^1^. An appropriate classification of hospitalized patients is warranted, in order to improve prognosis and allocation of resources.

Prediction and quantification of disease severity has been gaining attention during the pandemic. Disease severity assessments are generally based on clinical and biological parameters^2,3^. Some authors have proposed severity scoring and prediction based on imaging studies^4,5^ or a combination of both imaging and clinical data^6–8^. Computed tomography (CT) was used as the preferred imaging technique in the beginning of the pandemic in China for the diagnosis of patients with suspicion of COVID-19 (including negative reverse transcription polymerase chain reaction (RT-PCR) cases) and is useful for monitoring the disease progression^4^. Many algorithms have been published successfully identifying patients with COVID-19^9–11^ or automatically scoring disease severity based on CT findings^12–16^. However, despite significant inroads in artificial intelligence (AI) to the acquisition, segmentation and diagnosis of data in COVID-19^17^, little attention has been paid to automatic lesion subtyping. Parenchymal tissue lesions change during the progression and recovery from COVID-19 pneumonia^18,19^. Therefore, the automatic quantification and characterization of lesion subtypes based on deep learning may improve patient classification and management.

Recent studies have addressed the characterization of COVID-19 lung lesions using radiomic features. A seminal publication using CT data to predict outcomes focused on radiomic lobar analysis in order to characterize disease severity in a small cohort^20^. Other publications have proposed and compared several prediction models based on radiologist scores, CT whole lung radiomics and clinical variables^21^. The whole lung radiomic model may be superior to radiologists’ assessment predicting outcomes, disease severity and improving patient triage. Other authors have compared CT lesion radiomics, clinical and combined models to predict the progress and outcome of COVID-19^8^. More recently, Chassagnon et al. proposed an AI scheme for disease quantification, staging and outcome prediction using and ensemble of architectures^7^. The image features included the radiomic analysis of the COVID-19 related CT abnormalities as a single class as well as cardiopulmonary whole organ features. The image-based biomarkers were combined with clinical and biological variables in a severity and outcome prediction assessment.

Scarce attention has been paid to the design of severity and prognosis assessment models based on COVID-19 lung lesion subtyping. Only one prior work has proposed a deep learning model which differentiates between several classes^14^, other methods proposed postprocessing the resultant complete lesion segmentation through thresholding^22^ or the use of texture and intensity features^23^. In this context, this work contributes to the definition and comparison of COVID-19 prognosis prediction algorithms based directly on deep learning lesion subtyping from CT images. For this purpose, we specifically designed a deep learning algorithm, based on convolutional neural networks (CNN), for the automatic subtyping of COVID-19 compatible radiological patterns from CT images and the quantification of the extent of pulmonary involvement. Lesion characterization is used in order to assess disease severity and predict clinical outcomes, including mortality, admission to Intensive Care Units (ICU) and the need for mechanical ventilation. The proposed quantification and prediction models have been evaluated in a completely independent and multicentric cohort of 103 patients and compared to the radiologists’ assessment and a whole lung radiomics based model.

## Materials and methods

### Patient selection

This retrospective and multi-center study included 103 randomly selected, hospitalized patients diagnosed with COVID-19 confirmed by RT-PCR who underwent noncontrast chest CT for the assessment of disease severity at four different Spanish hospitals between March and July of 2020. The institutional review boards (IRBs) of all involved institutions approved this retrospective study (“Comité Ético de Investigación Clínica del Hospital Clínic de Barcelona”, “Comité Ético Hospital Universitario La Paz”, “Comité de Ética de la Investigación de la Universidad de Navarra”, “Comité Ético de Investigación Clínica, Instituto de Investigación Sanitaria – Fundación Jiménez Díaz” and “Comité de Ética de la Universidad Politécnica de Madrid”). Informed consent was obtained from all study participants. All experimental protocols complied with all relevant guidelines and regulations. Only CT acquisitions that were performed at the time of hospital admission were included in this study.

Chest CT examinations were performed using standard-of-care chest CT protocols in 12 different CT models from 5 different manufacturers. All CT exams were reconstructed with a 512 × 512 matrix and a slice thickness varying from 0.5 to 5 mm (mean of 1.5 mm). A deep inspiration breath-hold technique was performed whenever feasible. Acquisition details are shown in Supplementary Table S1.

Medical records were reviewed at each institution to collect clinical data including patient sex, age, mortality outcome (deceased or not deceased), ICU admission and length of stay, and the need for mechanical ventilation.

**Figure 1:**
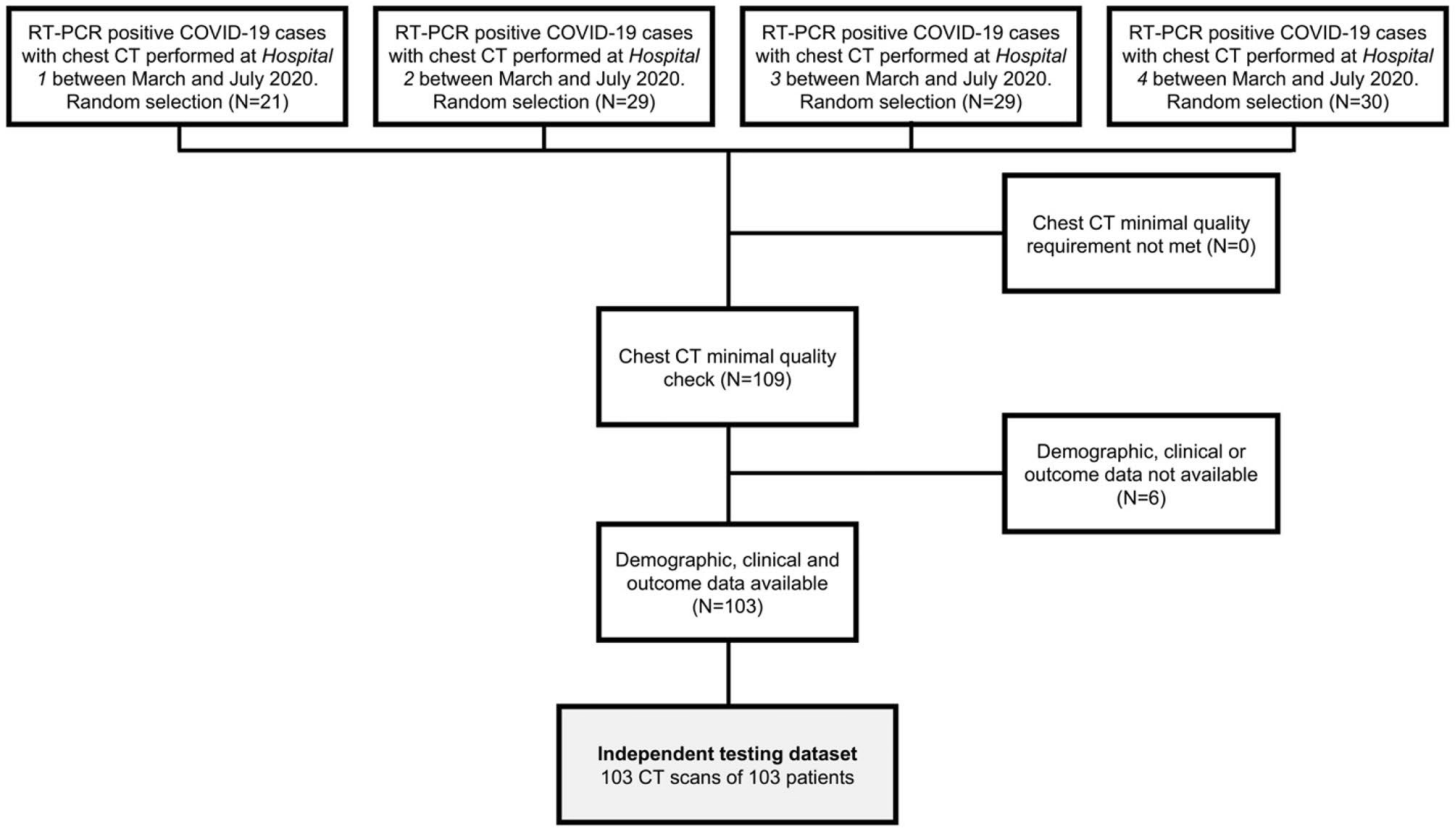
Flowchart shows patient inclusion in the study cohort used as independent testing for the lesion subtype segmentation algorithm as well as in the derived outcome prediction analysis.

All collected data were anonymized according to local guidelines. We assessed chest CT quality to discard scans with significant artefacts. Demographic and clinical data were obtained from digital records. Participants with missing demographic or clinical data were excluded (Fig. 1). Patient characteristics of the study cohort used as independent testing for the lesion subtype segmentation, severity assessment, and outcome prediction analysis are shown in Table 1.

**Table 1:** Characteristics of the testing and prediction study cohort.

Age (mean ± std) (years)	64.83 ± 13.96
Male	64 (62.14%)
Female	39 (37.86%)
Deceased	9 (8.7%)
Admitted to ICU	21 (20.3%)
Length of stay in ICU (mean ± std) (d)	3.06 ± 9.84
Needed mechanical ventilation	13 (12.61%)

N = 103. Note: data are numbers with percentages in parentheses or means ± standard deviations. y- years, d- days.

### Dataset for training the AI model

The dataset used for training the AI model for segmenting COVID19 lesion subtypes comes from a publicly available database collected and published by the Italian Society of Medical and Interventional Radiology. This database is composed of 100 axial CT slices belonging to 60 COVID-19 confirmed patients and were manually segmented by an expert radiologist considering two different types of parenchymal injuries, including ground glass opacities and consolidation. The images, which were in JPG format, were transformed to Hounsfield Units (HU) by establishing an intensity-transformation function from original values to HU considering the intensity values of air and fat areas.

### COVID-19 lesion subtype segmentation algorithm

**Figure 2:**
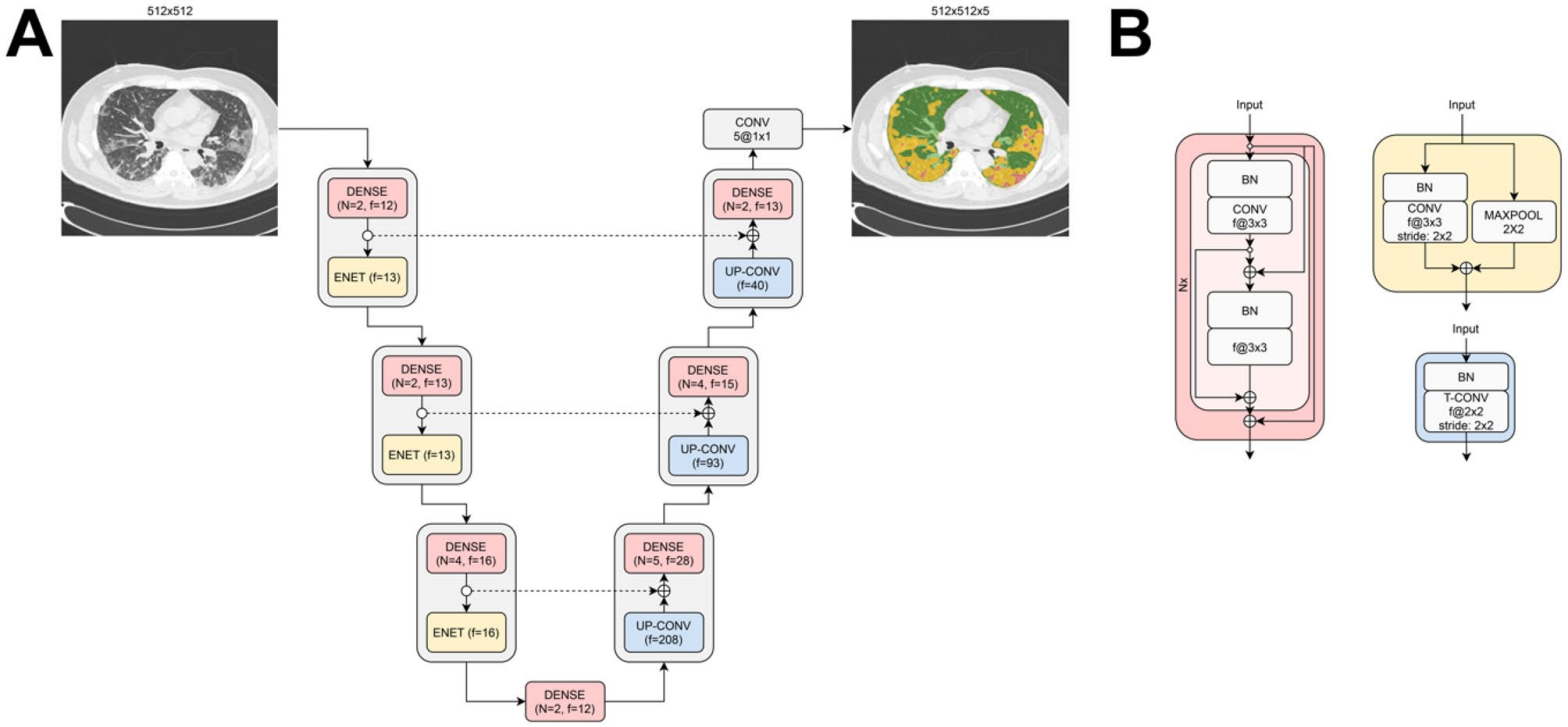
Deep learning COVID-19 lesion subtype segmentation architecture. (**A**) Dense-efficient deep learning architecture for the segmentation of parenchymal lesion subtypes in COVID-19 pneumonia. (**B**) Dense block used in both the encoding and decoding pathway of the architecture (red), ENET-style subsampling block (yellow) and transposed convolutional block (blue).

A 2D multiclass CNN-based segmentation architecture was specifically designed for segmenting COVID-19 radiographic subtypes. Our motivation to adopt a 2D architecture was based on our interest in analyzing independent 2D axial CT slices. This aspect of the approach makes it invariant to slice thickness, consequently, robust to the variability of scanning protocols across centers and vendors, Additionally, it allows training the network on annotated 2D images which is an advantage with respect to manual 3D annotations as they imply high burden to experts and are not always available. The network architecture is illustrated in Fig. 2. The encoder pathway is composed by encoder blocks. Each encoder block consists of a convolutional dense block which concatenates in an iterative manner previous feature maps from preceding layers. Dense connections alleviate the vanishing gradient problem and allow a stronger feature propagation throughout the network architecture, and thereby prevent the loss of informative features^24^. These dense blocks are subsequently down-sampled by an Efficient-Net block which combines the traditional max-pooling operation with strided convolutional operations to avoid representation bottlenecks and loss of information when performing the sub-sampling^25^. It has been previously shown that the integration of these blocks into pure 3D and 3D-to-2D segmentation architectures outperforms state-of-the-art CNN such as the well-known U-Net^26,27^. In the decoding pathway of the proposed architecture, transposed convolutional (deconvolutional) features together with higher resolution feature maps from the encoder, which are passed through skip-connections, are processed by a convolutional dense block. This decoding pathway ends with a convolutional operation with a kernel size of 1 × 1 producing 4 feature maps corresponding to the background, normal tissue, ground glass opacities and consolidation. The last convolutional operation has a softmax activation function which produces the final probability image map. The final image label map is then computed by assigning to each pixel in the image a label which has the highest probability. For the purpose of training the segmentation algorithm, we designed a class-weighted Dice-based loss function that accounts for class imbalance caused by differences in the prevalence of different COVID-19 lesion subtypes. We inversely weighed the loss function according to the number and size of the different classes in the training population to avoid frequency biases and to avoid discriminating against underrepresented COVID-19 lesions.

For optimizing the loss function, we used an Adam Stochastic Optimizer with an initial learning rate 1e-4 and plateau learning rate decay with a factor of 0.2 when the validation loss is not improved after 5 epochs. Training was terminated when the validation loss did not improve after 10 epochs. The model was trained from scratch and no transfer learning was used to initialize model parameters. The network was implemented in Keras with Tensorflow and using a PC with a GPU NVIDIA Quadro P6000 (24 GB). All CT scans were preprocessed by clipping the intensities outside the range [50, -1024] HU, and the remaining values were scaled between zero and one.

Two different experts (one radiologist and one expert in pulmonary imaging) manually edited the segmentation results obtained by the algorithm on a subset of CT scans using ITK-SNAP software (www.itksnap.org), and several 3D overlap and surface metrics were computed to evaluate the performance of the segmentation algorithm.

Additionally, all segmentation results were visually evaluated by experienced radiologists. This visual segmentation evaluation scoring was performed using scores which ranged from 0 to 10 according to the degree of under- or over- estimation of the different parenchymal subtypes (score 0: significant under-estimation, score 5: neither under- nor over-estimation, score 10: significant over-estimation).

### Evaluation of segmentation results

Automatic segmentation of disease subtypes using the AI model was performed in all 103 CT scans. Three chest radiologists with expertise in COVID-19 diagnosis and one expert in pulmonary imaging (MFV, CPM, GGM and MJLC, all with more than 10 years of experience in chest CT) participated in the revision of 3402 axial CT images covering full lung regions in 20 CT scans, including segmentation results obtained by the algorithm. Two independent readings were performed of this CT subset. The 20 CT scans were randomly selected making sure that all ranges of disease severity were included. The experts manually supervised and corrected all the segmentation label maps produced by the proposed algorithm. The Dice coefficient, the Hausdorff distance and the average surface distance for each parenchyma subtype was calculated to evaluate the similarity between the manual segmentation (manually corrected annotations) of each CT slice and automated segmentation generated by the AI model from a randomly selected set of 20 CT scans. The mean and the standard deviation were calculated.

Additionally, to make a complete the evaluation in all the testing cohort of 103 cases, the segmentation results obtained by the algorithm were visually evaluated by expert radiologists (MFV, CPM, GGM, GB, MS, MB) using a scoring scale from 0 to 10 to assess per case the degree of under- and over- estimation of each parenchymal subtype.

### Disease quantification

All test CT scans were preprocessed by segmenting and masking only the lungs’ regions using a robust and publicly available model for lung parenchyma segmentation^28^. Volumetric quantification of each parenchymal pattern, including healthy tissue, ground glass opacities and consolidation pattern areas, was performed in all CT scans after being processed by the COVID-19 segmentation algorithm. Relative percentage volume affected by each parenchymal pattern was calculated for each lung independently. Additionally, each lung was divided into three equivolumetric regions, and disease quantification was computed in each third. Disease extent was expressed as a percentage.

### Visual severity scoring of chest CT

An experienced radiologist per hospital, who was blinded to all patient information -including the three outcomes considered-, reviewed each CT scan recording the extent of COVID-19 lesions (ground glass and consolidations), total lung involvement, and the percentage of each pulmonary lobe (right upper, right middle, right lower, left upper, and left lower) affected. On CT scans, ground-glass opacity is defined as a hazy increased opacity of lung parenchyma, with preservation of bronchial and vascular margins and consolidation is a homogeneous increase in pulmonary parenchymal attenuation that obscures the margins of vessels and airway walls^29^. Visual severity scores based on lesion extension ranged from 1 to 5 for each subtype (score 1: < 5%, score 2: 5%-25%, score 3: 25%-50%, score 4: 50%-75%, score 5 > 75%)^30^. Additionally, total lung involvement (for each lung independently) was also scored.

### Patient prognosis

The automatic volumetric lesion quantification, performed by the AI algorithm, was used to assess patient severity and predict clinical outcomes. Relevant clinical outcomes included patient mortality, ICU admission and the need for mechanical ventilation. A total of 27 features were included in the regression model to predict clinical outcomes. These features included the total percentage of extension of each parenchymal subtype (normal parenchyma, ground glass opacities and consolidation pattern), relative percentage of each parenchymal subtype divided for each lung (right, left) and for each lung third (upper, middle, lower) and relative lesion involvement (combination of both ground glass and consolidation patterns) for each lung third.

The performance of our proposal was compared to the ability of the radiologist scores to predict the same clinical outcomes, and to the use of CT whole lung radiomic signatures which have been previously proposed for predicting ICU admission and patient outcome^21^. Signatures for predicting ICU admission included run entropy of the GLRLM, zone entropy of the GLSZM, large dependence low gray level emphasis of the GLDM and correlation of the GLCM. Signatures for predicting patient outcome were composed by cluster tendency of the GLCM, long run low gray level emphasis of the GLRLM, busyness of the NGTDM and large dependence low gray level emphasis of the GLDM. We used ICU admission signature for predicting the need for mechanical ventilation since no specific signature was proposed for this outcome.

Logistic regression models were used to evaluate the ability of extent-based parenchyma subtype features, whole lung radiomic features, and radiologist scores to predict patient outcomes using a five-fold cross validation strategy. The performance of each model was primarily evaluated by using the mean area under the ROC curve (AUC). Other metrics including sensitivity (SN), specificity (SP), positive predictive value (PPV) and negative predictive value (NPV) were also reported. Youden index was used to determine the optimal threshold. 95% confidence intervals (CIs) were calculated considering appropriate *t*-score and the estimated standard deviation computed with the cross-validation strategy.

## Results

### Evaluation of COVID-19 lesion subtype segmentation

The evaluation metrics of the segmentations results compared to the manually corrected annotations made by the experts in the subset of 20 CT scans are reported in Table 2. It should be noted that no significant differences in performance of the segmentation algorithm were observed among the different institutions.

**Table 2:** Quantitative evaluation of the COVID-19 lesion subtype segmentation algorithm.

Parenchyma subtype	Dice coefficient	Average surface distance (mm)	Hausdorff distance (mm)
Healthy tissue	0.985 (0.021)	0.106 (0.113)	1.143 (1.679)
Ground glass opacities	0.912 (0.158)	0.301 (0.381)	3.019 (5.542)
Consolidation	0.841 (0.254)	6.265 (26.813)	16.997 (44.453)

The table reports the average for each parenchymal pattern. Data in parenthesis are standard deviations. Both manual segmentations (radiologist and pulmonary imaging expert) were considered.

Regarding the visual evaluation of the segmentation results of the total cohort composed by 103 CT scans, the mean absolute error, defined as the distance to 5 (neither under- nor over- estimation) was 0.55 ± 0.64, 0.81 ± 0.82 and 0.97 ± 1.14 for normal tissue, ground glass opacities and consolidation pattern respectively. Means of underestimation (scores ≤ 5) were 4.40 ± 0.65, 4.37 ± 0.65, 4.25 ± 0.65 while means of overestimation (scores ≥ 5) were 5.6 ± 0.69, 5.74 ± 0.68, 5.94 ± 1.25 for each subtype respectively.

**Figure 3:**
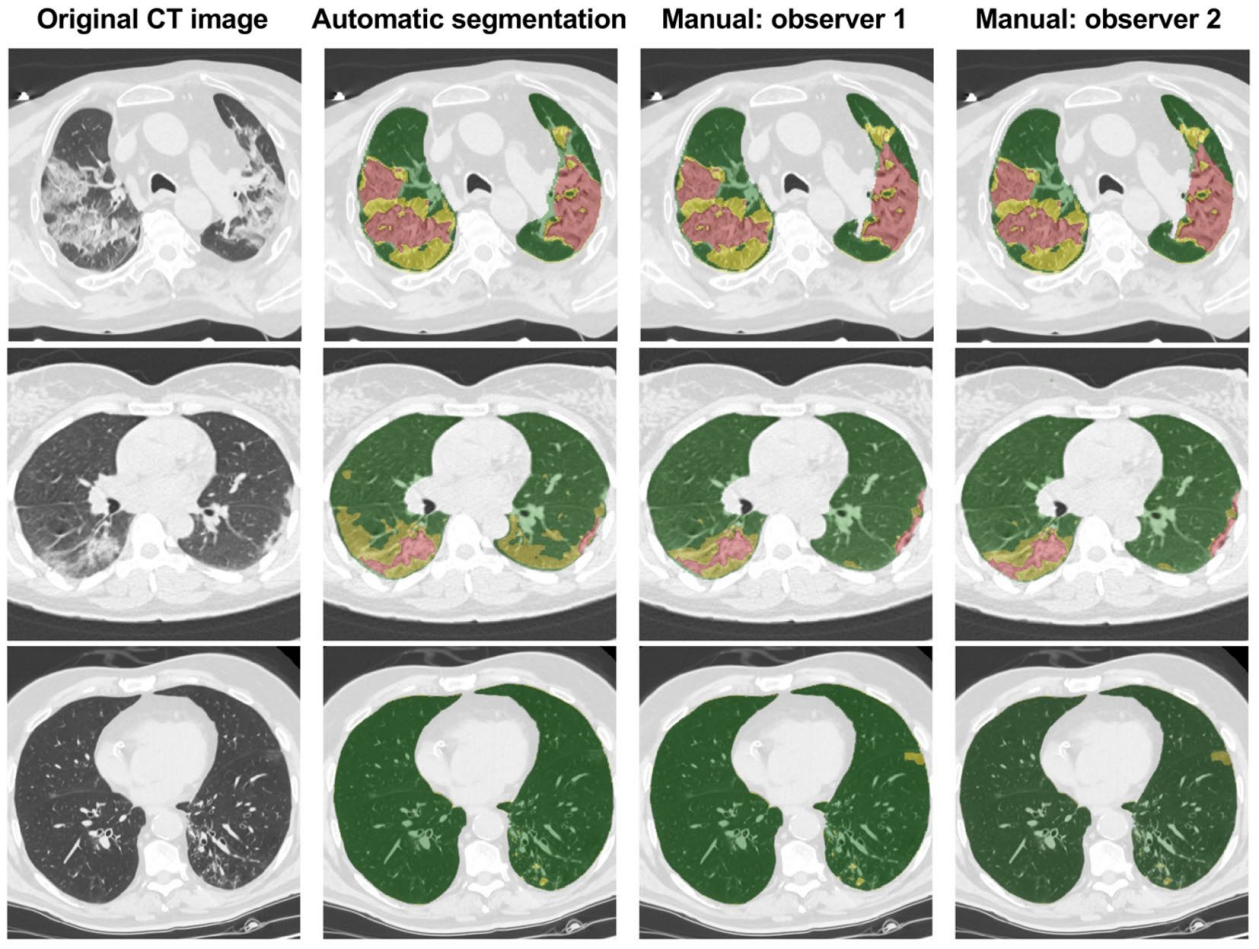
Coronavirus Disease 2019 (COVID-19) lesion subtyping results of three cases with different parenchymal involvement (from top to bottom, 74 year old man, 59 year old woman, 69 year old man, from extensive to little involvement). Nonenhanced CT scans in the axial view (left column) overlaid with the automatic lesion subtyping segmentation (second column) and the corrected scans by the two observers (3rd and 4th columns) are shown. Colors correspond to healthy parenchyma (green), ground glass opacities (yellow) and consolidation (reddish).

The most common errors identified were the overestimation of ground glass opacities and consolidation lesions in basal zones, caused by motion artifacts as well as confusion with pleural effusion areas. Figure 3 shows the corrections made to the results produced by the algorithm performed by the experts in 3 cases with different parenchymal involvement.

### Disease quantification

**Figure 4:**
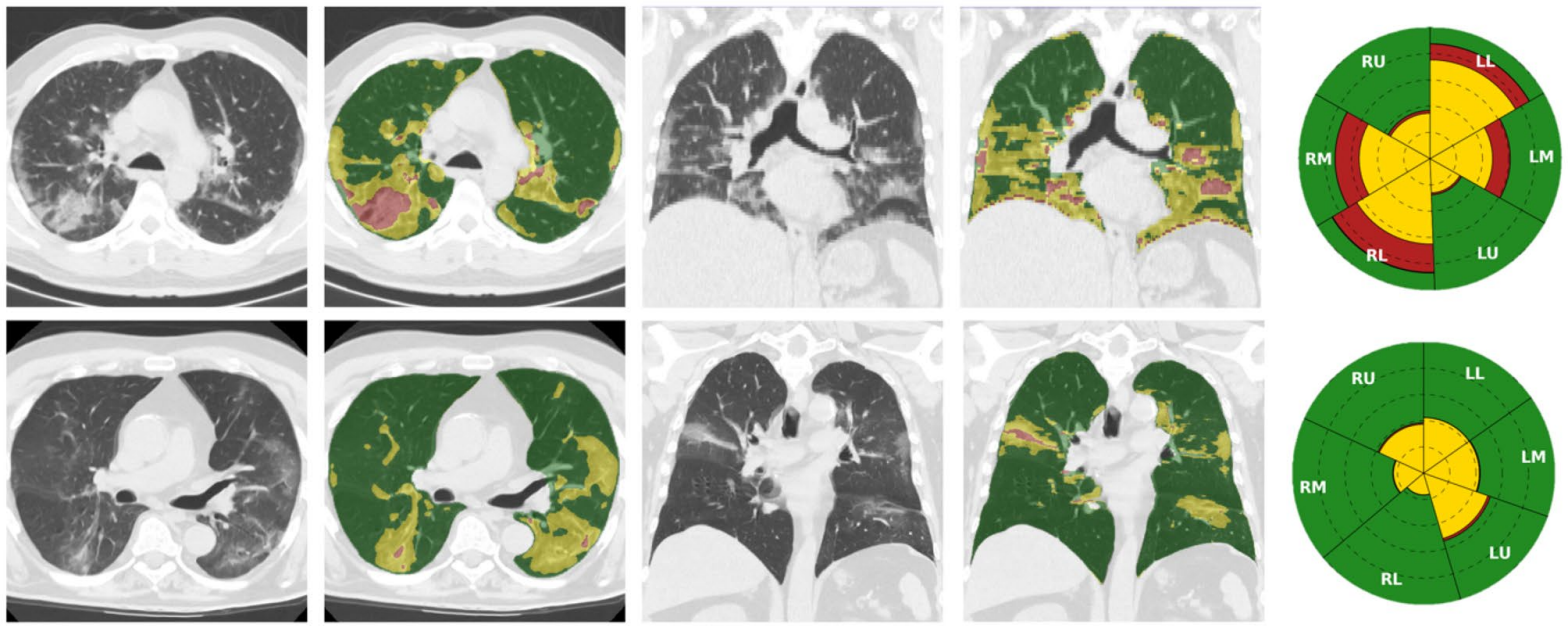
Automatic disease quantification. Nonenhanced CT axial and coronal views (first and third columns) overlaid with the automatic lesion subtyping segmentation (second and fourth columns) of two cases. Colors of the overlay correspond to normal parenchyma (green), ground glass (yellow), consolidation (reddish). First case of a 55 year old man with moderate lung involvement (upper row), and another case of a 71 year old man with mild lung involvement (lower row). The corresponding glyphs (right column) show the percentage of lung zones for each subtype shown in colors: healthy parenchyma (green), ground glass opacities (yellow) and consolidation (red). Lungs’ regions represented in the glyphs correspond to: RU- right lung upper region, RM- right lung middle region, RL- right lung lower region, LU- left lung upper region, LM- left lung middle region, LL- left lung lower region.

The proposed method is an attempt at a valid and practical tool for radiologists to quantify disease severity. The algorithm automatically generates a report in an easy-to-read PDF format (see supplementary Figure S1), including the lung involvement COVID-19 disease subtypes. This solution provides detailed quantification of disease subtypes by reporting the percentage of affected volume for each whole lung and lung zone with respect to the total lung volume. The report also includes a visual glyph representation which sums up the volume metrics, allowing intuitive estimates of the affected areas at diagnosis and during patient follow-up. Figure 4 shows two cases, including moderate and mild lung involvement. The corresponding glyphs are shown on the right.

**Figure 5:**
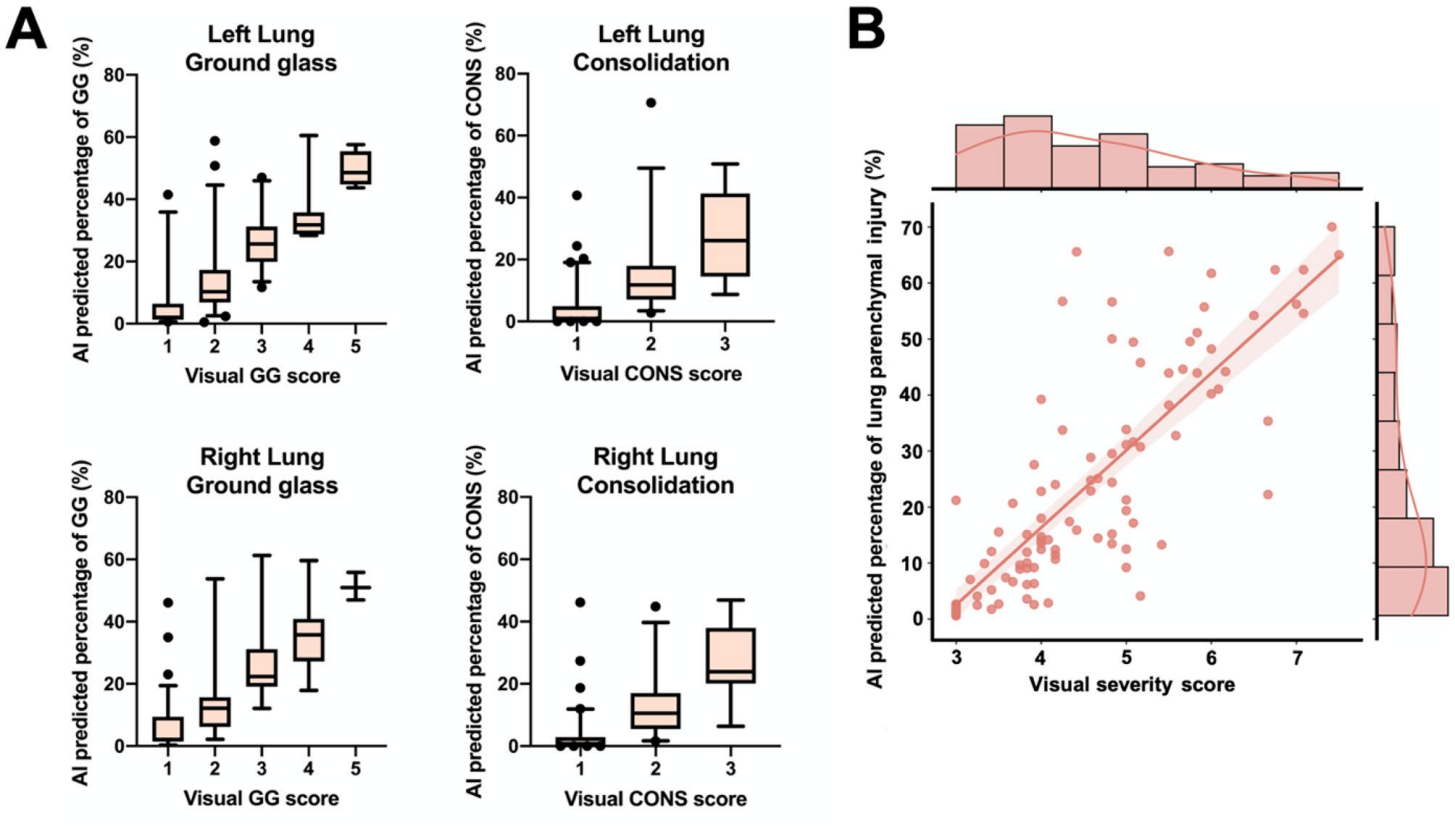
Disease severity assessment. A: boxplots representing the relationship between the automatic AI-predicted percentage of each lesion subtype and the severity scores visually determined by radiologists. The horizontal line in each box illustrates the median, and the whiskers represent 5th and 95th percentiles. B: relation between visually and automatically defined CT severity score considering total lesion involvement. Visual severity scores ranged from 1 to 5 for each subtype (score 1: < 5%, score 2: 5%-25%, score 3: 25%-50%, score 4: 50%-75%, score 5 > 75%).

The segmentation algorithm was executed in the entire test cohort, and lung involvement of disease subtypes was calculated for all cases. Figure 5a shows the relation between the AI-predicted percentage of each disease subtype and the radiologists’ score. A good agreement between them was found, although the assessment overestimated disease severity compared to the AI-base prediction. It should be noted that visual assessment of lesion involvement is a subjective procedure, and it has been previously shown that visual readings tend to overestimate the extent of the disease^31^. The agreement between the percentage of the total affected lung tissue with respect to the total severity score assigned to each patient thought visual inspection was also assessed (Fig. 5b). The total visual severity score was calculated as the sum of the scores for each subtype. Visual scoring correlated well with predicted total percentage of lesion, with a Spearman correlation coefficient (R) of 0.83 (95% CI: 0.755, 0.884).

### Patient prognosis

Twenty-one patients in our study were admitted to ICU, thirteen required mechanical ventilation and nine died during hospitalization.

Regression models including the features based on the automatic quantification of parenchyma subtypes predicted mortality with an AUC of 0.874 (95% CI: 0.790, 0.959), ICU admission with an AUC of 0.726 (95% CI: 0.582, 0.871), and mechanical ventilation with an AUC of 0.679 (95% CI: 0.496, 0.862) (Table 3). Automatic and objective measurements of lung lesion volumes performed better than radiologist based visual scoring (Table 3). Simple features such as automatically detected volumes of parenchymal subtypes were also better outcomes predictors when compared to more complex CT radiomic features. The results presented in Table 3, show a slight tendency of the proposed method to overestimate the different clinical outcome, having higher PPV values, without decreasing the specificity of the system. We consider this is a useful feature in prognostic models for clinical decision guidance, especially in the context of the management of patients with COVID-19, as it is necessary to grant surveillance and proper management to all the potential severe cases and it is not desirable to miss any of them. Most prominent features for all three outcomes prediction models were the relative percentage of normal parenchyma and consolidation pattern for each lung third. Percentage of normal tissue had a negative weight in the model while there was a strong positive relation between the percentage of consolidation pattern and all clinical outcomes. The relative percentages of ground glass opacities had also a positive relation with the outcomes in the prediction models, however with a lower impact than that obtained for the percentage of consolidation pattern.

**Table 3:** Performance analysis of prediction models based on DL-based lesion subtyping, full lung radiomics or radiologist assessment for three outcomes (mortality, ICU admission and need of mechanical ventilation) studied using five-fold cross validation in a cohort of 103 subjects with RT-PCR positive COVID-19 pneumonia.

Outcome	Features	AUC [95% CI]	SN [95% CI]	SP [95% CI]	PPV [95% CI]	NPV [95% CI]
Mortality	DL-based lesion subtyping	0.874 [0.790,0.959]	1 [1]	0.775 [0.674,0.876]	0.5167 [0.196,0.838]	0.4833 [0.162,0.804]
	Radiomics (full lung)	0.838 [0.749,0.927]	0.917 [0.791,1]	0.862 [0.78,0.944]	0.25 [−0.13,0.63]	0.5 [0.062,0.938]
	Radiologist	0.725 [0.620,0.830]	0.875 [0.685,1]	0.7 [0.52,0.88]	0.25 [-0.13,0.63]	0.5 [0.062,0.938]
ICU admission	DL-based lesion subtyping	0.726 [0.582,0.871]	0.867 [0.743,0.991]	0.638 [0.475,0.801]	0.3462 [-0.0118,0.704]	0.4167 [0.0367,0.797]
	Radiomics (full lung)	0.624 [0.446,0.802]	0.758 [0.61,0.906]	0.588 [0.37,0.806]	0.3235 [-0.0345,0.681]	0.6875 [0.329,1.05]
	Radiologist	0.543 [0.394,0.691]	0.517 [0.248,0.78]	0.783 [0.63,0.936]	0.1111 [-0.0579,0.28]	0.6389 [0.279,0.999]
Mechanical ventilation	DL-based lesion subtyping	0.679 [0.496,0.862]	0.938 [0.843,1]	0.579 [0.453,0.705]	0.25 [-0.13,0.63]	0.75 [0.093,1.41]
	Radiomics (full lung)	0.675 [0.494,0.857]	0.917 [0.791,1]	0.549 [0.359,0.739]	0.125 [-0.065,0.315]	0.875 [0.685,1.06]
	Radiologist	0.302 [0.110,0.494]	0.917 [0.791,1]	0.192 [0.01,0.394]	0 [0,0]	1 [1]

AUC, SN, SP, PPV, NPV and 95% confidence intervals are reported for each model.

## Discussion

Artificial intelligence can provide tools capable of estimating COVID-19 disease severity and predicting clinically relevant outcomes such as mortality, ICU admission, and the need for mechanical ventilation. CT findings in the lung of infected patients are one of the earliest indicators of disease. Therefore, the quantification of each disease subtype may play an important role in the management of COVID-19 patients.

We hereby report our findings using an AI system to automatically segment lung lesion subtypes in COVID-19 pneumonia from CT images. Our results demonstrate that AI is a valid tool for the identification and quantification of lesion subtypes in COVID-19 pneumonia (Dice coefficient of 0.985 ± 0.02-healthy tissue, 0.912 ± 0.15-ground-glass, 0.84 ± 0.25-consolidations and visual assessment confirmed no relevant under- or over-estimation with mean absolute error of 0.55 ± 0.64-healthy tissue, 0.81 ± 0.82-ground glass and 0.97 ± 1.14-consolidations), and its results are associated with the visually determined presence of parenchymal injuries and disease severity as assessed by radiologists (Spearman correlation coefficient (R) of 0.83 (95% CI: 0.755, 0.884)). Furthermore, the use of simple metrics in prediction models of relevant outcomes outperforms whole lung radiomic models or radiologists scoring for predicting the aforementioned clinically relevant outcomes, with an AUC of 0.874 (95% CI: 0.790, 0.959), 0.726 (95% CI: 0.582, 0.871) and 0.679 (95% CI: 0.496, 0.862) for predicting mortality, admission to ICU and the need for mechanical ventilation respectively. An extended performance analysis using different algorithms to predict these clinical outcomes is presented in Table S3.

Parenchymal lung disease subtyping has been previously used to characterize emphysema, interstitial lung abnormalities and interstitial lung disease using deep learning as well as other histogram based local methods^15,32–34^. Despite the abundance of COVID-19 manuscripts published to date, few studies have focused on lesion subtyping^17^. Our results not only demonstrate the efficacy of COVID-19 lesion subtyping using deep learning techniques, but also its potential role in patient stratification and predicting different outcomes.

Previous works had address disease severity assessments based on clinical and biological parameters^2,3^, imaging studies^4,5^ or a combination of both imaging and clinical data^6,7,14^. Yue et al.^20^ defined a prediction model of hospital stay using CT data using radiomics from lung lobes. Homayounieh et al.^21^ proposed and compared several prediction models based on radiologist scores, CT whole lung radiomics, and clinical variables. In our work we compared our deep learning approach with their CT whole lung radiomics model. Lesion subtyping demonstrated superiority in terms of AUC with respect to whole lung radiomics in the three outcomes considered. The results in our cohort of the whole lung radiomics model are comparable to those reported in the original publication.

Chassagnon et al.^7^ proposed an AI scheme including radiomic lesion features and cardiopulmonary whole organ features with clinical and biological variables in their severity and outcome prediction assessment. In comparison to this work our approach presented a similar performance in terms of AUC with a simpler image processing approach and without including clinical variables. The integration of clinical variables in our models is expected to improve prediction accuracy as has been previously reported in other works^14,20^. Zhang et al.^14^ focused mostly their work on differential diagnosis but they also proposed a critical illness prediction model considering different lesion subtypes, extracted using a 2D deep learning model with seven classes, as well as clinical parameters. Critical illness was defined as the combined outcome of ICU admission, mechanical ventilation or death. As shown in our work lesion subtypes were identified as the most relevant variables in their prediction model. Performance in terms of AUC of their model based on lesion features are comparable to the ones found in our work. In this work they did not compare the performance with respect to the predictor based on the radiologist scores nor considered differentiated outcomes.

Therefore, in the context of the previous literature we highlight the following strengths. Our work confirms of the importance of lesion subtyping in prognosis analysis and the proposed prediction model based in deep learning segmentation shows high AUC scores for several clinical outcomes, specifically mortality and admission to ICU. The completely independent multicentric evaluation with data from 12 different scanner models confirms the robustness of the segmentation model with respect to the variability introduced by medical device. The quantitative segmentation evaluation shows very high Dice coefficient for the segmentation of the ground glass opacity regions.

Our study had several limitations. First, regarding the training cohort, only axial slices from 60 patients were available limiting the exploitation of the three-dimensional nature of the CT datasets. This multicentric dataset was selected as it was one of few resources for COVID-19 lesion subtype labeling during the early days of the pandemic, including ground glass, consolidations pattern and pleural effusion areas. Our system was designed and ready to include the three lesion subtypes. Pleural effusions could not be evaluated given their low prevalence in the training dataset and in the study cohort. To date, this is the only publicly available dataset that we are aware of that includes labeling of pleural effusion, evidencing a clear need to the community to provide CT datasets with accurate and complete labeling of COVID-19 lesion subtypes (see supplementary Table S2 for an up to date list of publicly available datasets). As presented in the results section, the consideration of pleural effusion subtype as a separate class in the AI system is important as it may lead to overestimation of consolidation patterns, and more importantly it would enable the quantification and follow-up of the pleural effusion with important implications for the patient management.

Our deep learning architecture is two-dimensional, as we prioritized that the method would be invariant to slice thickness, and consequently, robust to the variability of scanning protocols across centers and vendors. We acknowledge that spatial consistency would improve, and basal segmentation could be better tackled by using a three-dimensional approach. However, the proposed lesion subtype segmentation of axial slices performed well for whole lung assessment and was also considered as an advantage in time constrained scenarios when only few slices need to be evaluated. Another limitation is related to the evaluation of the segmentation technique that was quantitatively performed for a subset of images (20 cases) and visually for all the testing cohort following an overestimation and subestimation score for each lesion subtype. Additionally, scoring was regional as opposed to lobal, and therefore not anatomical. Automatic lobar segmentation is very challenging in diseased lungs, and we preferred to ensure a robust partition to avoid biasing the results. Recent COVID-19 studies have successfully demonstrated accurate lobar segmentation^12^ and the integration with the presented AI system would be certainly feasible and could be considered in the future.

A final limitation could be related to the number of cases in the training and testing cohorts. As previously commented, the training database was one of the only databases available at the beginning of the pandemic with subtype labeling. Although, the number of slices and patients included could be considered low, recently it has been demonstrated that semantic segmentation properly configured can be trained effectively with low numbers of cases^35^, that is consistent with the evaluation results of our proposal in the independent testing cohort from four different hospitals. Similarly, the testing cohort could be considered as having a low number of cases. In this sense, as data collection is an intense labor task, we prioritized the multicentric nature of the cohort to ensure sufficient variability (see supplementary Table S1 for acquisition details) to evaluate the results for both segmentation and outcome prediction models. Considering publicly available datasets, supplementary Table S2 presents an updated list after a careful literature review. Only two additional datasets^14,36^ include subtypes labeling in a limited number of cases or slices that could be considered for extending the training cohort of the presented segmentation algorithm, but given the good evaluation results it doesn’t seem necessary. On the other hand, no dataset includes subtyping and detailed outcomes information including ICU stay, need for mechanical ventilation and mortality hampering the extension of the testing cohort for both segmentation and outcome prediction tasks. Despite the relevant efforts in data collection and algorithm development during the pandemic, there is still a clear need of complete COVID-19 databases that would include CT imaging data, lesion subtyping labeling, clinical data covering demographics, symptoms and hemogram based biomarkers, as well as sufficient follow-up information and differentiated clinical outcomes. Only when this data is available a robust comparison of diagnostic and prognostic algorithms and methods in large cohorts could be performed.

The main implication of our study is the demonstration of use of deep learning techniques to automatically characterize COVID-19 pneumonia lung lesion subtypes to better predict mortality and admission to ICU and at a lower extent the need for mechanical ventilation. Participating radiologists in this study confirmed the validity of the designed quantification and prediction tools (including the automatic reporting) and expressed an interest in using them in their routine clinical practice during the pandemic. Moreover, further studies using our methodology could enable an objective and quantitative understanding of disease progression and response to therapies, as well as the objective evaluation of drug efficacy in clinical trials.

In conclusion, our study demonstrates that an AI system can identify COVID-19 lung lesion subtypes in nonenhanced CT scans with a performance comparable to expert radiologists scoring. Lesion subtyping enables a better stratification and risk assessment of patients based on the prediction of clinically relevant outcomes.

## Supplementary Information

Supplementary Information.
