# Supplementary material for: Deep learning-based lesion subtyping and prediction of clinical outcomes in COVID-19 pneumonia using chest CT
Source: Sci Rep. 2022 Jun 7;12:9387. doi: 10.1038/s41598-022-13298-8 (PMC9172615; doi:10.1038/s41598-022-13298-8)
Supplement: Supplementary file 1 — Supplementary Information. [file 41598_2022_13298_MOESM1_ESM.pdf]

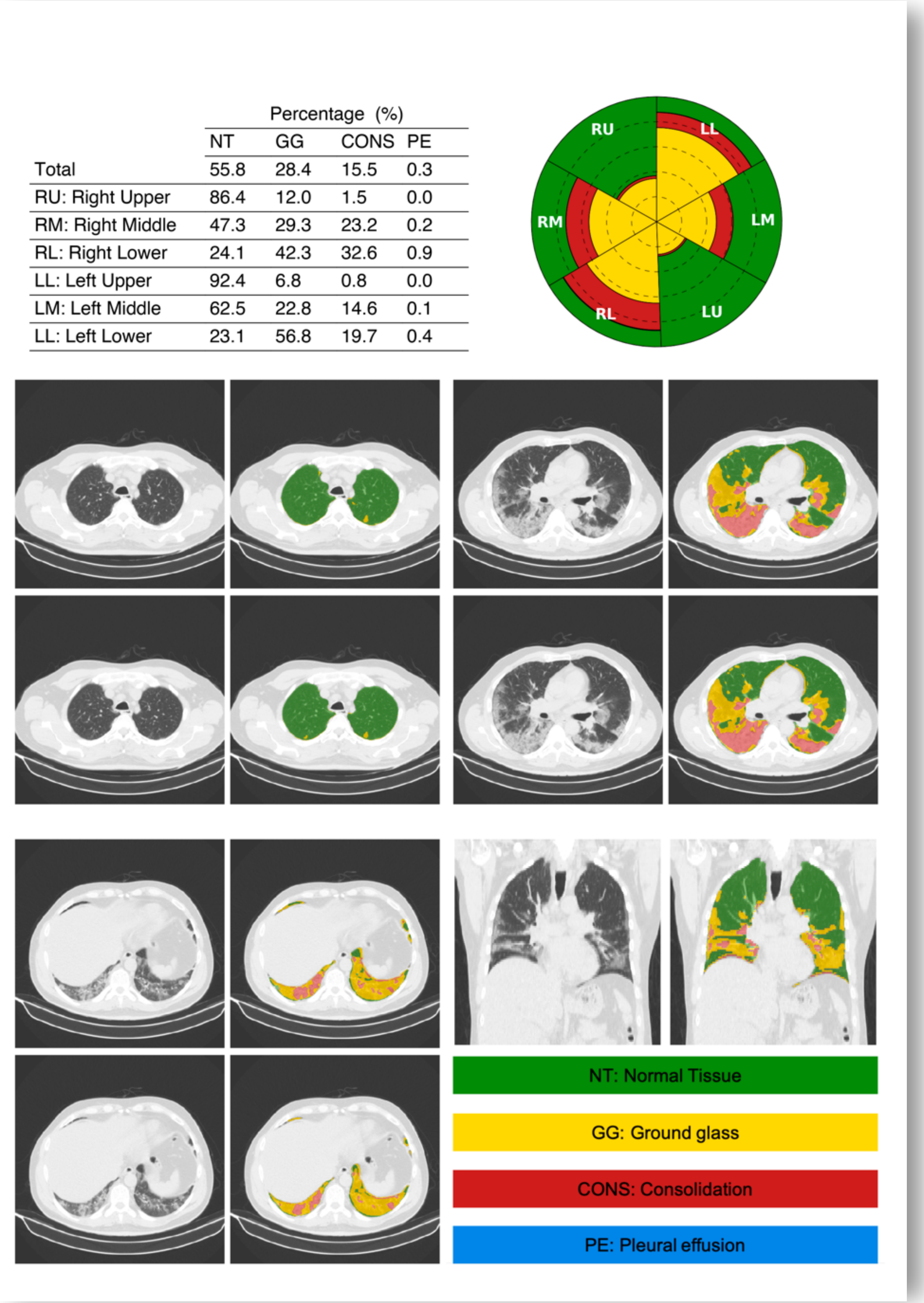

	Percentage (%)			
	NT	GG	CONS	PE
Total	55.8	28.4	15.5	0.3
RU: Right Upper	86.4	12.0	1.5	0.0
RM: Right Middle	47.3	29.3	23.2	0.2
RL: Right Lower	24.1	42.3	32.6	0.9
LL: Left Upper	92.4	6.8	0.8	0.0
LM: Left Middle	62.5	22.8	14.6	0.1
LL: Left Lower	23.1	56.8	19.7	0.4

Figure 1S. Example of the PDF report automatically generated for one subject with moderate lung involvement.

Table 1S. CT acquisition parameters per model. Data in parenthesis are interquartile ranges (IQR).

Manufacturer	Model	KVP	Exposure time (ms)	Tube Current (mA)	N
Agfa	ClinApps	120 (120-120)	500 (500-500)	369 (369-369)	1
Canon	Aquilion Prime SP	110.5 (100-120)	350 (350-350)	261.1 (152-373)	19
Philips	Brilliance 64	120 (120-120)	1978.5 (435.2-4500)	232.5 (30-371.25)	15
Siemens	Biograph64	100 (100-100)	500 (500-500)	643 (643-643)	1
Siemens	Emotion 16	110 (110-110)	600 (600-600)	242.8 (202.7-281)	21
Siemens	Emotion 16 (2010)	130 (130-130)	600 (600-600)	165.8 (127-140)	3
Siemens	SOMATOM Definition Flash	111.2 (100-120)	812.8 (285-500)	548.6 (194-1048)	16
Siemens	SOMATOM Drive	95 (92.5-97.5)	500 (500-500)	769 (756.5-781.5)	2
Siemens	SOMATOM X.cite	100 (100-100)	250 (250-250)	841.6 (790-945)	3
Siemens	SOMATOM go.Up	128.5 (130-130)	869.7 (866.7-941)	184.1 (163.1-213)	12
Siemens	Sensation 64	120 (120-120)	500 (500-500)	540.5 (532-567.2)	9
Toshiba	Aquilion	120 (120-120)	500 (500-500)	440 (440-440)	1

Table 2S. Publicly available COVID-19 CT datasets published to date.

Dataset	Description	Manual segmentation of COVID-19 lesion subtypes	Clinical data	Clinical outcomes	Number patients
https://www.qmenta.com/blog/covid-19-ct-segmentation-dataset	This is a dataset of 100 axial CT images from the Italian Society of Medical and Interventional Radiology's excellent collection of about 60 patients with Covid-19 that were converted from openly accessible JPG images.	Yes	No	No	100 images from 60 patients
https://radiopaedia.org/articles/covid-19-3	This dataset contains axial volumetric chest CTs	No	No	No	101 patients
https://bit.ly/BSTICovid19_Teaching_Library	This dataset is an imaging database of known UK patients	No	Yes, demographic data, PCR result, temperature at admission, blood test result	No	18 patients
https://github.com/UCSD-AI4H/COVID-CT	This dataset is a collection of open source CT images of COVID-19 cases from preprint articles	No	Yes, demographic data, medical history	No	349 images from 216 patients
https://zenodo.org/record/3757476	This dataset contains labeled COVID-19 CT scans. Left lung, right lung, and infections are labeled by two radiologists and verified by an experienced radiologist.	No subtypes, unique lesion label	No	No	20 patients
https://mosmed.ai/datasets/covid19_1110	This dataset contains anonymised human lung computed tomography (CT) scans with COVID-19 related findings, as well as without such findings.	No subtypes, unique lesion label. Only in 50 patients	No	No	1110 patients
https://github.com/BIMCV-CSUSP/BIMCV-COVID-19	Dataset with CT imaging of COVID-19 patients along with their radiographic findings, pathologies, PCR, immunoglobulin G (IgG) and immunoglobulin M (IgM) diagnostic antibody tests and radiographic reports	Yes, only in 10 images	Yes, demographic data, diagnostic tests (PCR, IgG, IgM)	No	163 patients
K Zhang et al. http://ncov-ai.big.ac.cn/download?lang=en	Dataset of the CT images and metadata are constructed from cohorts from the China Consortium of Chest CT Image Investigation (CC-CCII).	Yes, only in 750 CT slices	Yes, demographic data, liver function, lung function	Only critical illness	3777 patients
Afshar et al https://doi.org/10.6084/m9.figshare.12991592	Covid-CT-MD: COVID-19 computed tomography (CT) scan dataset applicable in machine learning and deep learning.	No	Yes, demographics data, PCR result, symptoms	Only hospitalization in 17 patients	307 patients

Table 3S. Performance analysis of different prediction models based on DL-based lesion subtyping, whole lung radiomics, and radiologists' assessment for three clinical outcomes (mortality, ICU admission, and need for mechanical ventilation). The mean and 95% confidence interval of the area under the ROC curve (AUC) for each model are reported.

Outcome	Features	**Linear Regression**	Decission Tree	Random Forest	KNN	SVM
Mortality	DL-based	**0.8742 [0.79, 0.959]**	0.5829 [0.437, 0.729]	0.8664 [0.765, 0.967]	0.7382 [0.628, 0.848]	0.4226 [-0.0334, 0.879]
	Radiomics	0.838 [0.749, 0.927]	0.7074 [0.672, 0.743]	0.8016 [0.619, 0.985]	0.8694 [0.788, 0.951]	0.3582 [0.0452, 0.671]
	Radiologist	0.7247 [0.621, 0.829]	0.7234 [0.487, 0.959]	0.8614 [0.73, 0.992]	0.7635 [0.502, 1.02]	0.5762 [0.208, 0.944]
ICU admission	DL-based	**0.7263 [0.582, 0.87]**	0.4765 [0.38, 0.573]	0.6903 [0.506, 0.874]	0.6015 [0.539, 0.664]	0.4629 [0.187, 0.739]
	Radiomics	0.6241 [0.446, 0.802]	0.5696 [0.456, 0.684]	0.652 [0.466, 0.838]	0.6176 [0.442, 0.794]	0.6017 [0.408, 0.796]
	Radiologist	0.5404 [0.392, 0.688]	0.4972 [0.384, 0.61]	0.5589 [0.403, 0.715]	0.4773 [0.28, 0.674]	0.3908 [0.274, 0.508]
Mechanical ventilation	DL-based	**0.6787 [0.496, 0.862]**	0.5226 [0.434, 0.612]	0.6358 [0.452, 0.82]	0.6001 [0.366, 0.834]	0.5332 [0.352, 0.714]
	Radiomics	0.6753 [0.494, 0.856]	0.5124 [0.374, 0.65]	0.6514 [0.472, 0.83]	0.623 [0.509, 0.737]	0.476 [0.096, 0.856]
	Radiologist	0.3022 [0.11, 0.494]	0.4321 [0.352, 0.513]	0.3814 [0.175, 0.587]	0.414 [0.258, 0.57]	0.443 [0.14, 0.746]
